# Effectiveness of Seasonal Influenza Vaccines against Influenza-Associated Illnesses among US Military Personnel in 2010–11: A Case-Control Approach

**DOI:** 10.1371/journal.pone.0041435

**Published:** 2012-07-31

**Authors:** Angelia A. Eick-Cost, Katie J. Tastad, Alicia C. Guerrero, Matthew C. Johns, Seung-eun Lee, Victor H. MacIntosh, Ronald L. Burke, David L. Blazes, Kevin L. Russell, Jose L. Sanchez

**Affiliations:** 1 Armed Forces Health Surveillance Center (AFHSC), Silver Spring, Maryland, United States of America; 2 US Air Force School of Aerospace Medicine (USAFSAM), 711^th^ Human Performance Wing, Wright Patterson Air Force Base, Ohio, United States of America; 3 Henry M. Jackson Foundation for the Advancement of Military Medicine, Inc., (HJF), Bethesda, Maryland, United States of America; University of Hong Kong, Hong Kong

## Abstract

**Introduction:**

Following the 2009 influenza A/H1N1 (pH1N1) pandemic, both seasonal and pH1N1 viruses circulated in the US during the 2010–2011 influenza season; influenza vaccine effectiveness (VE) may vary between live attenuated (LAIV) and trivalent inactivated (TIV) vaccines as well as by virus subtype.

**Materials and Methods:**

Vaccine type and virus subtype-specific VE were determined for US military active component personnel for the period of September 1, 2010 through April 30, 2011. Laboratory-confirmed influenza-related medical encounters were compared to matched individuals with a non-respiratory illness (healthy controls), and unmatched individuals who experienced a non-influenza respiratory illness (test-negative controls). Odds ratios (OR) and VE estimates were calculated overall, by vaccine type and influenza subtype.

**Results:**

A total of 603 influenza cases were identified. Overall VE was relatively low and similar regardless of whether healthy controls (VE = 26%, 95% CI: −1 to 45) or test-negative controls (VE = 29%, 95% CI: −6 to 53) were used as comparison groups. Using test-negative controls, vaccine type-specific VE was found to be higher for TIV (53%, 95% CI: 25 to 71) than for LAIV (VE = −13%, 95% CI: −77 to 27). Influenza subtype-specific analyses revealed moderate protection against A/H3 (VE = 58%, 95% CI: 21 to 78), but not against A/H1 (VE = −38%, 95% CI: −211 to 39) or B (VE = 34%, 95% CI: −122 to 80).

**Conclusion:**

Overall, a low level of protection against clinically-apparent, laboratory-confirmed, influenza was found for the 2010–11 seasonal influenza vaccines. TIV immunization was associated with higher protection than LAIV, however, no protection against A/H1 was noted, despite inclusion of a pandemic influenza strain as a vaccine component for two consecutive years. Vaccine virus mismatch or lower immunogenicity may have contributed to these findings and deserve further examination in controlled studies. Continued assessment of VE in military personnel is essential in order to better inform vaccination policy decisions.

## Introduction

Influenza virus infections are very common in the military, mostly due to conditions facilitating their spread such as crowded living conditions, stressful working environments and deployment-associated exposures [1]–[4]. Respiratory infections are responsible for 25 to 30 percent of both outpatient illness and hospitalizations among US military personnel [5] and pneumonia and influenza result in hundreds of hospitalizations annually in the military [6], [7].

Trivalent inactivated vaccines (TIV) have been in use by the US military for the past six decades [8] and the live-attenuated influenza vaccine (LAIV) was added during the 2003–04 influenza season. LAIV has been used primarily among recruits undergoing initial entry training and young military personnel; where it has been found to provide better protection against influenza-associated illnesses [9]. By contrast, military-based studies have suggested that TIV is more efficacious against laboratory-confirmed influenza among highly-immunized military personnel [10].

A recent study evaluating seasonal influenza VE against pandemic (pH1N1) virus associated illnesses in 2009–10 [11] noted better protection with TIV (44%) than with LAIV (24%) among highly-immunized military personnel as well as a “priming” effect by preceding immunization which was associated with a significantly increased degree of protection (eg, 41% higher). Using case-control analytic approaches, several estimates of 2009–10 and 2010–11 seasonal influenza VE have been published to-date, mostly in European Union populations where VE has ranged from 42% to 72% [12]–[18]. More recently, early VE estimates for 2011–12 season (December to February) have been found to be 55% in Spain [19] and 43% in the I-MOVE network of seven countries [20] against prevailing A/H3 virus. No similar data, however, have been published for US-based populations for the 2010–11 or 2011–12 seasons. Thus, we sought to provide an end-of-season assessment of the effectiveness of the 2010–11 seasonal influenza vaccines against clinically-apparent, laboratory-confirmed, influenza illnesses. Our collaborators at USAFSAM will also be publishing separately an analysis of mid-season and end-of-season VE of the 2011–12 seasonal vaccines. Similar case-control DoD estimates of VE, including subtype-specific estimates, will continue to be conducted on an annual basis to guide future military vaccination policy decisions.

## Materials and Methods

The surveillance population of interest was all active component service members who served at some point during the period of September 1, 2010 through April 30, 2011. Data were obtained from the Defense Medical Surveillance System (DMSS), a large relational database maintained at the Armed Forces Health Surveillance Center (AFHSC) which contains longitudinal data including demographic characteristics, occupations, immunizations and medical encounters for US military personnel [21]. Additionally, data on laboratory-confirmed cases and test-negative controls were obtained from a Department of Defense influenza reference laboratory at the United States Air Force School of Aerospace Medicine (USAFSAM) [22] and represented cases detected at US military treatment facilities in the United States and internationally.

Cases were defined as active component service members with a laboratory-confirmed influenza-associated illness, detected by reverse transcriptase polymerase chain reaction (RT-PCR) or viral culture methods. Cases were identified through one of the service-specific notifiable disease reporting systems [23] or by collaborating investigators at USAFSAM [22]. Since cases provided by USAFSAM were identified by a nasal wash or nasopharyngeal (NP) swab specimen submitted to the laboratory, they were additionally required to have an inpatient or outpatient medical encounter occurring within seven days of the specimen collection date in order to be considered a case. An individual was eligible to be a case only once during the study period; if an individual had more than one case-defining diagnosis, then only the first episode was included. Nasal wash/NP samples were mostly taken within 72 hours of symptom onset as per DoD influenza surveillance recommendations. For cases reported via service-specific notifiable disease reporting systems, respiratory illnesses that met a clinical case definition (sudden onset of fever >102.2°F, respiratory symptoms and either myalgia or headache) and were laboratory-confirmed as influenza were evaluated.

Two groups were selected for comparison, 1) a test-negative control group which consisted of individuals suffering from non-influenza respiratory illnesses, and 2) a healthy control group, consisting of individuals who had a medical encounter for a non-respiratory illness within three days of the case’s medical encounter. Healthy controls were required to have a musculoskeletal (International Classification of Diseases, Ninth Revision, Clinical Modification (ICD-9-CM) 700–739, 810–848, or V54) or mental health encounter (ICD-9-CM 700–739, 810–848, or V54) with no documented respiratory problems (ICD-9-CM 001–139, 320–326, 380–382, 460–519, 780.6, 780.7, 786, or 787.0 ) during the medical visit. In order to control for baseline immunity, gender and geographic influenza distribution, healthy controls were additionally matched to cases on age, sex, and location. Individuals in both control groups were excluded if they had an inpatient, outpatient, or reportable medical event encounter at any point during the study period with a diagnosis of influenza (ICD-9-CM 487 or 488). A maximum of four controls were matched to each case.

Immunization data from DMSS were used to determine whether cases and controls received any influenza vaccination during the period of September 1, 2010 through April 30, 2011. Subjects who received an influenza vaccine at least 14 days prior to the date of their qualifying medical encounter were considered immunized; all others (those immunized less than 14 days prior to or after the medical encounter, or those not vaccinated with any of the 2010–11 seasonal influenza vaccines) were considered non-immunized for the purposes of this evaluation. Additionally, immunization data from DMSS for the period of 2004–05 through 2009–10 seasons was also obtained in order to control for prior immunization history, an important potential confounder in our highly-immunized military [11]. Non- immunization of personnel may be due to their non-availability due to deployment, medical contraindications (such as history of hives or other significant allergic reactions to previous influenza vaccination) or simply due to non-availability of vaccine at the unit, clinic or hospital medical level.

Crude odds ratios (OR) were calculated for comparison of cases to controls by multiple factors including sex, age group (<25 years, 25 to 29 years, 30 to 39 years, 40 years and over), race-ethnicity (White, Black, Hispanic, Asian/Pacific Islander, American/Alaskan Indian, Other/unknown), Branch of service (Army, Air Force, Coast Guard, Navy, Marine Corps), hospitalization status, location at diagnosis (US versus non-US), month of diagnosis, and any prior influenza vaccinations since 2004.

Adjusted odds ratios (AOR) for vaccination status were calculated using logistic and conditional logistic regression (depending on whether the cases and controls were matched). The test-negative control analysis adjusted for sex, age group, number of prior vaccinations and month of diagnosis. The healthy control analysis adjusted for sex, age group, and number of prior vaccinations. VE was defined as (1–AOR)*100. Vaccine type (TIV versus LAIV) and influenza subtype (A/H3, A/H1 and B) specific analyses were also conducted. The subtype analysis was restricted to USAFSAM cases and healthy control subjects due to availability of data. All analyses were performed using SAS 9.1.3 (SAS Institute, Cary, North Carolina, USA).

This study was reviewed by the US Air Force Research Laboratory (AFRL) Institutional Review Board and was determined not to constitute human use research according to the 32 CRD 219.102 (d) (AFRL IRB number: FWR20110097N). The opinions and assertions contained herein are solely those of the authors and do not reflect the official policy or position of the US Department of Defense (DoD) or of its subordinate services (Army or Air Force) medical authorities.

## Results

During the period of September 1, 2010 to April 30, 2011, a total of 603 individuals meeting the case definition were identified; 177 (29.4%) from the USAFSAM data and 426 (70.6%) from the reportable medical events data (Tables 1 and 2). The majority of cases were male (71.8%), White (59.2%), from the Air Force (46.8%), and vaccinated at least once prior to 2010–11 season (88.4%). Case subjects were similar to healthy controls with the exception of sex (71.8% vs. 77.3% males; OR = 0.74, 95% CI: 0.60 to 0.91) and vaccination prior to 2010–11 season (88.4% vs. 96.3% previously immunized, respectively; OR = 0.24, 95% CI: 0.16 to 0.35) (Table 1). Compared to test-negative controls, cases were statistically more likely to be female, older, Black or Hispanic, in the Army, and previously vaccinated for influenza (Table 2). Of note, we detected a clear difference in the monthly occurrence of cases vs. controls during the season; namely, 81% of test-positive cases occurred in the January-April 2011 timeframe compared to only 55% of test-negative controls. Cases and controls were found to be similarly distributed with respect to location (US = 83%, Europe = 6%, other international locations = 11%).

**Table 1:** Characteristics of Influenza Cases Matched to Healthy Controls.

Characteristic	Cases	Controls	Crude odds ratio
	n (%)	n (%)	(95% CI)
Sex			
Male	410 (71.8)	1766 (77.3)	**0.74 (0.60–0.91)**
Female	161 (28.2)	518 (22.7)	Ref
Age group			
<25	175 (30.6)	621 (27.2)	Ref
25–29	153 (26.8)	545 (23.9)	0.99 (0.77–1.26)
30–39	184 (32.2)	725 (31.7)	0.89 (0.70–1.12)
40+	59 (10.3)	393 (17.2)	**0.51 (0.37–0.72)**
Race-ethnicity			
White	338 (59.2)	1439 (63.0)	Ref
Black	105 (18.4)	439 (19.2)	1.01 (0.79–1.30)
Hispanic	56 (9.8)	215 (9.4)	1.11 (0.81–1.53)
Asian/Pacific Islander	31 (5.4)	93 (4.1)	1.45 (0.94–2.23)
American Indian/Alaskan Native	6 (1.0)	30 (1.3)	0.84 (0.35–2.04)
Other/unknown	35 (6.1)	68 (3.0)	2.21 (1.44–3.39)
Service			
Army	232 (40.6)	943 (41.3)	Ref
Air Force	267 (46.8)	1063 (46.5)	1.16 (0.74–1.80)
Marine Corps	17 (3.0)	93 (4.1)	0.61 (0.27–1.39)
Navy	49 (8.6)	165 (7.2)	1.46 (0.84–2.55)
Coast Guard	6 (1.0)	20 (0.9)	1.46 (0.46–4.68)
Number of prior vaccinations			
0	66 (11.6)	84 (3.7)	Ref
1+	505 (88.4)	2200 (96.3)	**0.24 (0.16–0.35)**

Bolded cells indicate statistical significance.

**Table 2:** Characteristics of Influenza Cases and Unmatched Test-Negative Controls.

Characteristic	Cases	Controls	Crude odds ratio
	n (%)	n (%)	(95% CI)
Sex			
Male	432 (71.6)	356 (80.5)	**0.61 (0.45–0.82)**
Female	171 (28.4)	86 (19.5)	Ref
Age group			
<25	181 (30.0)	210 (47.5)	Ref
25–29	159 (26.4)	89 (20.1)	**2.07 (1.49–2.87)**
30–39	195 (32.3)	113 (25.6)	**2.00 (1.47–2.72)**
40+	68 (11.3)	30 (6.8)	**2.63 (1.64–4.22)**
Race-ethnicity			
White	358 (59.4)	316 (71.5)	Ref
Black	107 (17.7)	56 (12.7)	**1.69 (1.18–2.41)**
Hispanic	62 (10.3)	20 (4.5)	**2.74 (1.62–4.63)**
Asian/Pacific Islander	32 (5.3)	19 (4.3)	1.49 (0.83–2.67)
American Indian/Alaskan Native	6 (1.0)	11 (2.5)	0.48 (0.18–1.32)
Other/Unknown	38 (6.3)	20 (4.5)	1.68 (0.96–2.94)
Service			
Army	237 (39.3)	25 (5.7)	Ref
Air Force	282 (46.8)	374 (84.6)	**0.08 (0.05–0.12)**
Marine Corps	19 (3.1)	16 (3.6)	**0.12 (0.06–0.27)**
Navy	53 (8.8)	25 (5.7)	**0.22 (0.12–0.42)**
Coast Guard	12 (2.0)	2 (0.4)	0.63 (0.13–2.99)
Number of prior vaccinations			
0	68 (11.3)	88 (19.9)	Ref
1+	535 (88.7)	354 (80.1)	**1.96 (1.39–2.76)**
Month of diagnosis			
September	18 (3.0)	41 (9.3)	–
October	19 (3.1)	52 (11.8)	–
November	21 (3.5)	53 (12.0)	–
December	58 (9.6)	55 (12.4)	–
January	174 (28.9)	76 (17.2)	–
February	199 (33.0)	74 (16.7)	–
March	95 (15.7)	68 (15.4)	–
April	19 (3.1)	23 (5.2)	–

Bolded cells indicate statistical significance.

Overall VE was relatively low and similar regardless of whether healthy controls (VE = 16%, 95% CI: −1 to 45) or test-negative controls (VE = 29%, 95% CI: −6 to 53) were used as comparison groups (Table 3), with borderline statistical significance. VE for the TIV vaccine was 23% (95% CI: −1 to 42) and 53% (95% CI: 25 to 71) for the healthy control and test-negative control analyses, respectively. In contrast, VE estimates for LAIV vaccine were 11% (95% CI: −15 to 31) and −13% (95% CI: −77 to 27) for the healthy control and test-negative control analyses, respectively. Although the VE was higher for TIV recipients compared to LAIV recipients for both the healthy and test-negative control analyses, this difference was not found to be statistically significant.

**Table 3:** Influenza Vaccine Effectiveness by Control Group and Vaccine Type.

		Cases	Controls	Crude odds ratio	Adjusted odds	Vaccine Effectiveness
		n (%)	n (%)	(95% CI)	ratio (95% CI)*	(95% CI)
**Healthy controls (matched)** *						
Vaccinated (any vaccine)						
Yes		458 (80.2)	1903 (83.3)	**0.81 (0.53–0.96)**	0.84 (0.55–1.01)	16% (−1 to 45)
No		113 (19.8)	381 (16.7)	Ref	Ref	Ref
Vaccinated (TIV)						
Yes		151 (57.2)	687 (64.3)	**0.74 (0.56–0.97)**	0.77 (0.58–1.01)	23% (−1 to 42)
No		113 (42.8)	381 (35.7)	Ref	Ref	Ref
Vaccinated (LAIV)						
Yes		301 (72.7)	1163 (75.3)	0.87 (0.68–1.11)	0.89 (0.69–1.15)	11% (−15 to 31)
No		113 (27.3)	381 (24.7)	Ref	Ref	Ref
**Test-negative controls (unmatched)^**						
Vaccinated (any vaccine)						
Yes		485 (80.4)	308 (69.7)	1.79 (1.34–2.38)	0.71 (0.47–1.06)	29% (−6 to 53)
No		118 (19.6)	134 (30.3)	Ref	Ref	Ref
Vaccinated (TIV)						
Yes		170 (59.0)	168 (55.6)	1.15 (0.83–1.59)	**0.47 (0.29–0.75)**	**53% (25 to 71)**
No		118 (41.0)	134 (44.4)	Ref	Ref	Ref
Vaccinated (LAIV)						
Yes		307 (72.2)	139 (50.9)	2.51 (1.82–3.45)	1.13 (0.73–1.77)	−13% (−77 to 27)
No		118 (27.8)	134 (49.1)	Ref	Ref	Ref

* Adjusted for sex, age group, and number of prior vaccinations.

^Adjusted for sex, age group, number of prior vaccinations, and month of diagnosis.

Subtype-specific VE estimates revealed differing protection depending on the influenza subtype responsible for the illness (Table 4). Although subtype-specific data was only available for USAFSAM cases, there were still 77, 61 and 33 A/H3, A/H1, and B cases identified, respectively. Non-pandemic A/H1 cases were not identified during the 2010–11 season. A moderate level of protection against A/H3 was found (VE = 58%, 95% CI: 21 to 78). By contrast, VE against subtype B was found to be lower, but not statistically significant (VE = 34%, 95% CI: −122 to 80). Of note, the analysis did not find any protection against A/H1 for either vaccine type, although this was not statistically-significant (VE = −38%, 95% CI: −211 to 39). Overall, VE for any vaccination was found to be 37% (95% CI: −10 to 64).

**Table 4:** Influenza Vaccine Effectiveness by Influenza Subtype: Comparison to Matched Healthy Controls.

Influenza Subtype	Vaccinated	Cases	Controls	Odds ratio	Vaccine Effectiveness
		n (%)	n (%)	(95% CI)	(95% CI)
**Influenza A/H1 (pandemic)**					
	Yes	53 (86.9)	202 (82.8)	1.38 (0.61–3.11)	−38% (−211 to 39)
	No	8 (13.1)	42 (17.2)	Ref	Ref
**Influenza A/H3**					
	Yes	59 (76.6)	273 (88.6)	0.42 (0.22–0.79)	**58% (21 to 78)**
	No	18 (23.4)	35 (11.4)	Ref	Ref
**Influenza B**					
	Yes	29 (87.9)	121 (91.7)	0.66 (0.20–2.22)	34% (−122 to 80)
	No	4 (12.1)	11 (8.3)	Ref	Ref

* Influenza subtype data only available for USAFSAM cases. Due to small sample size, adjusted estimates not available.

## Discussion

The results of this assessment suggest there is a low to moderate degree of protection against A/H3 and B, but not against A/H1 strains that circulated in the US during the 2010–11 season. The low VE against clinically-apparent, laboratory-confirmed influenza illnesses among active component US military service members is somewhat unexpected. However, this is the first study among a primarily US-based population to report VE estimates for the 2010–11 season and may end up being comparable as other data are released on the general US population.

VE estimates for the 2010–11 [12]–[18] and early for 2011–12 season [19], [20] have been reported for the European Union (EU). Although the overall estimates of VE in this study are somewhat lower than those reports from the EU, when the findings are restricted to TIV VE compared to test-negative controls (a more appropriate comparison as the vaccines used in the EU studies are inactivated vaccines), the results are more similar. A study by Kissling et al reported adjusted VE for eight EU states to be 52% overall and 41% for the 15 to 59 year age group [15]. Similar estimates were also reported by Steens et al for the Netherlands (46%) and by Savulescu et al for Spain (50%), both of which used a test-negative control comparison group [13], [14]. Contrary to our findings of no VE for the A/H1 subtype, Kissling et al reported a VE of 27% for A/H1 among 15 to 59 year olds, however, this did not reach statistical significance [15].

There are a number of factors that may have played a role in the low to moderate VE estimates found in this study. There is the potential that the vaccine viruses were a mismatch with the circulating viruses. This has been reported in some previous seasons and has resulted in low VE [24], [25]. Although isolates from the general US population reported by the CDC for the 2010–11 season indicated a close match between the circulating and vaccine viruses [26], this genetic drift could have occurred later in the season and perhaps among strains which circulated among military personnel [27]. This may be especially true for the A/H1 strains where there was an apparent lower immunogenicity and protection provided by vaccines among recruits as described by Myers et al [27]. An additional study by US military collaborators at the US Naval Health Research Center which investigated the genetic characteristics of the A/H1 viruses that circulated in the military recruit population during the 2010–11 season and associated comparisons of the immune responses generated by LAIV and TIV vaccines in this same population is in the publication stage. Noteworthy to mention, however, is the fact that this study has found modest amino acid differences in circulating strains compared to the vaccine strain and could provide much needed answers to these questions (personal communication, Commander Patrick Blair).

Lower than expected VE may also be due to population factors. The military population is highly immunized against influenza, typically at greater than 90% [28]; while the US civilian population of a similar age range (18 to 49 years) has overall vaccination rates of no more than 40% [29]. Previous studies have found decreased VE among highly immunized military populations, especially for the LAIV vaccine, but higher LAIV VE among vaccine-naïve populations, such as military recruits [9]–[11]. For this study, stratification of VE by vaccine type revealed lower and non-significant VE for LAIV recipients compared to TIV. Since almost twice as many of our cases received LAIV compared to TIV, this difference in vaccine type VE may help to explain the overall finding of lower than expected VE in this population. The case-control design of this study may also partially explain the overall lower than expected VE estimates. A simulation model by Ferdinands and Shay, found that case-control studies of VE underestimate true VE by as much as 11.9%, principally due to biases introduced by the lack of diagnostic specificity of tests used (not a factor in our study since we based our cases on RT-PCR and/or culture diagnosis) [30]. All of these explanations warrant additional investigation, perhaps using populations with varying immunization rates and controlled cohort-based studies, to confirm and better understand the mechanisms at play. In addition, VE estimates need to be examined with relation to the degree of severity of influenza-associated illnesses, that is to say, comparison for hospitalized (more severe) versus non-hospitalized outcomes.

One important factor which we could control for was the sensitivity of the influenza-detecting assays given that their sensitivity are known to decrease over time (eg, lower sensitivity of RT-PCR and culture after 48 to 72 hours of illness). In our study, time from symptom onset to specimen collection did not differ between test-positive and test-negative cases (median = 2 days for both groups). Thus, there should have been no difference in influenza detection between test-positive and test-negative cases, given this very narrow sampling window.

There are several strengths and limitations to this study. The use of laboratory-confirmed, clinically-diagnosed influenza cases strengthens this study by providing a more specific case definition. A second strength is the use of both “healthy” and “test-negative” controls for comparison, which provided different methodologies to account for potential biases that can occur in case-control studies of VE [31]. The military population also provides a robust population to study VE as they represent a relatively healthy, young-to-middle aged adult population that is sometimes overlooked in other VE studies. Additionally, medical encounters and vaccines have near complete capture electronically for all active component personnel.

Of note, it is difficult to directly compare the healthy control population to the test-negative control population because the healthy controls were matched to the cases based on demographic characteristics. However, prior history of vaccination does appear to be different between the two control populations. The test-negative controls were more similar to the cases with regards to prior vaccination history (80% with one or more prior influenza vaccinations) than the healthy control population (96%). This probably reflects evidence of better health care seeking behavior and/or opportunities for prior vaccination in healthy controls, thus, comparisons using test-negative controls may represent a more appropriate comparison population in the military population for this and future influenza VE case-control studies.

One important limitation is the fact that the military population is highly immunized, thus, the results of this study may not be generalizable to the general US population. The study was also limited by the number of influenza cases that were laboratory-confirmed. There were probably many more influenza cases that occurred among military personnel, but not all were laboratory-confirmed or sought medical attention. If the cases selected for laboratory confirmation were different from other influenza cases, perhaps due to severity of illness, then the findings may be biased and may not be generalizable to all influenza infections occurring in the military. There may also be unknown biases and confounders that were not accounted for in the adjusted models.

In conclusion, a low level of protection against clinically-apparent, laboratory-confirmed, influenza-associated illness was found for the 2010–11 seasonal influenza vaccines in this military population. TIV immunization was associated with higher protection than LAIV, however, no protection against A/H1 was noted, even though a pandemic virus strain was a vaccine component for the second year in a row. These findings may provide justification towards preferential use of inactivated vaccines as a primary option for “seasoned” (eg, highly-immunized) US military personnel**.** Continued future annual assessments of influenza vaccine efficacy and/or effectiveness are necessary in the military setting in order to better guide vaccination policies and influenza infection control efforts.
